# Demographic consequences of greater clonal than sexual reproduction in *Dicentra canadensis*

**DOI:** 10.1002/ece3.2163

**Published:** 2016-05-10

**Authors:** Chia‐Hua Lin, Maria N. Miriti, Karen Goodell

**Affiliations:** ^1^ Department of Evolution, Ecology and Organismal BiologyThe Ohio State University318 West 12th AvenueColumbusOhio43210; ^2^ Department of Evolution, Ecology and Organismal BiologyThe Ohio State University1179 University DriveNewarkOhio43055; ^3^ Present address: Department of EntomologyThe Ohio State University– Ohio Agricultural Research and Development Center1680 Madison AvenueWoosterOhio44691

**Keywords:** Asexual reproduction, between‐ramet geitonogamy, loop analysis, matrix population model, plant population and community dynamics, reproductive assurance, self‐incompatible breeding system, spring ephemeral, tuber

## Abstract

Clonality is a widespread life history trait in flowering plants that may be essential for population persistence, especially in environments where sexual reproduction is unpredictable. Frequent clonal reproduction, however, could hinder sexual reproduction by spatially aggregating ramets that compete with seedlings and reduce inter‐genet pollination. Nevertheless, the role of clonality in relation to variable sexual reproduction in population dynamics is often overlooked. We combined population matrix models and pollination experiments to compare the demographic contributions of clonal and sexual reproduction in three *Dicentra canadensis* populations, one in a well‐forested landscape and two in isolated forest remnants. We constructed stage‐based transition matrices from 3 years of census data to evaluate annual population growth rates, *λ*. We used loop analysis to evaluate the relative contribution of different reproductive pathways to *λ*. Despite strong temporal and spatial variation in seed set, populations generally showed stable growth rates. Although we detected some pollen limitation of seed set, manipulative pollination treatments did not affect population growth rates. Clonal reproduction contributed significantly more than sexual reproduction to population growth in the forest remnants. Only at the well‐forested site did sexual reproduction contribute as much as clonal reproduction to population growth. Flowering plants were more likely to transition to a smaller size class with reduced reproductive potential in the following year than similarly sized nonflowering plants, suggesting energy trade‐offs between sexual and clonal reproduction at the individual level. Seed production had negligible effects on growth and tuber production of individual plants. Our results demonstrate that clonal reproduction is vital for population persistence in a system where sexual reproduction is unpredictable. The bias toward clonality may be driven by low fitness returns for resource investment in sexual reproduction at the individual level. However, chronic failure in sexual reproduction may exacerbate the imbalance between sexual and clonal reproduction and eventually lead to irreversible loss of sex in the population.

## Introduction

Many flowering plants can reproduce both sexually by seeds and clonally by propagules derived from vegetative organs. The ability to switch facultatively between sexual and clonal reproduction can be advantageous when local resources are spatially heterogeneous (Gardner and Mangel 1999) or in populations with low pollination success (van Groenendael et al. 1996; Aigner 2004). Although clonal offspring may be dispersal‐limited, they are typically more robust due to greater maternal resources and thus are better competitors than seedlings (Cook 1985; Ronsheim 1996; Silvertown 2008). Clonality also contributes to genet fitness through increased potential for future reproduction (Pan and Price 2002; Mizuki et al. 2005). Reproductive strategy, then, has direct bearing on the ecology and evolution of plants.

Resource allocation theory predicts trade‐offs between sexual and clonal reproduction at the individual level. Investments in sexual functions, such as production of flowers, gametes, and seeds, are often negatively correlated with investments in clonal offspring (Rameau and Gouyon 1991; Fischer and van Kleunen 2002; Obeso 2002) and can reduce future individual growth and reproduction by depleting energy reserves (Snow and Whigham 1989; Agren and Willson 1994; Primack and Stacy 1998). Empirical studies have shown that removing meristematic modules of clonal growth stimulates flower production, while increasing sexual reproduction reduces clonal reproduction in many species (Hartemink et al. 2004; Thompson and Eckert 2004; Liu et al. 2009; van Drunen and Dorken 2012). Likewise, reduction in sexual fecundity resulted from inadequate pollination may promote clonal reproduction, which may serve as a type of “reproductive assurance” that allows the genet to proliferate even when sexual reproduction fails (Nagylaki 1976).

The costs of clonality‐dominant reproductive strategies are also apparent at the population level. Extensive clonal reproduction often results in spatial aggregation of clone mates due to frequent short‐distance dispersal of clonal propagules. This aggregation can interfere with sexual reproduction by reducing the chance of receiving outcross pollen (Widen 1992; Charpentier 2002; Honnay et al. 2006). Clonal aggregation may also hinder seedling establishment because seeds not dispersing away from the patch suffer intense intraspecific competition with ramets (Eriksson 1992). Consequently, extensive clonal reproduction combined with inadequate sexual reproduction can further reduce successful outcrossing by increasing geitonogamy and suppressing seedling growth.

The negative consequences of excessive clonal reproduction on sexual reproduction can be intensified in isolated, fragmented habitats, or in founder populations colonizing new habitats. In self‐incompatible plants, the frequency of compatible mates continues to diminish by genetic drift after the population becomes isolated, further inhibiting sexual reproduction (Wagenius et al. 2007). Loss of genetic variation caused by extensive clonal reproduction is expected to reduce a population's ability to respond to selective pressures imposed by changing environmental conditions. Aside from leaving a population more vulnerable to stochastic decline, extensive clonal reproduction can cause recombination to be permanently lost, resulting in an “extinction debt,” in which clonal reproduction only prolongs a population's time to extinction (Honnay and Bossuyt 2005; Silvertown 2008).

An observed bias toward clonality could reflect a plant's strategies to maximize fitness (Ceplitis 2001; Vallejo‐Marin et al. 2010), or be a consequence of inadequate sexual reproduction (Eckert 2002; Honnay and Bossuyt 2005). Distinguishing between these alternatives in natural populations can improve our understanding of the ecological context of plant reproductive strategies and help predict how populations may respond to changes in resource availability and biotic interactions.

In this study, we studied the population dynamics of three distinct populations of *Dicentra canadensis* (Goldie) Walp, an understory herb in the temperate deciduous forests of eastern North America. Using field census data, we calculated population growth rates and quantified the contribution of clonal and sexual reproductive strategies to population growth. We then examined the population‐ and individual‐level consequences of these strategies. At the population level, we compared the growth rates of populations with different reproductive strategies. We also explored how increased pollination and no‐pollination scenarios would affect population growth. At the individual level, we asked: Do trade‐offs exist between clonal and sexual reproduction that could limit investment in one or the other reproductive strategy? Specifically, we measured the degree to which sexual reproduction influences patterns of resource allocation. We integrated individual‐ and population‐level effects of reproductive strategy by simulating the demographic responses to changes in sexual reproductive strategy.

## Methods

### Study species and sites

*Dicentra canadensis* is a spring ephemeral found in the understory of old‐growth, mesic, deciduous forests of eastern North America. Because of its specific habitat associations, it is considered unlikely to establish populations in secondary forests (Bratton et al. 1994). In late March, shortly after snowmelt, *D. canadensis* emerge aboveground, quickly grow, and reproduce before the deciduous trees fully leaf out. At emergence, each plant consists of a primary tuber produced in the previous year, shallow roots, and one photosynthetic leaf (Appendix S1a). Plants accumulate biomass at the base of the leaf stalk to form a new tuber, which eventually becomes the plant's energy reserve for the following year. Large plants may produce additional, secondary tubers that serve as clonal propagules with the capacity to form new ramets the following year (Appendix S1b,c). Some large plants also produce single inflorescences holding 3–8 flowers in early–mid April (Appendix S1b,d). The flowers stay fresh for 3–7 days and are pollinated by bumble bee queens (*Bombus* spp.) foraging for nectar. Seeds of *D. canadensis* mature in May and are dispersed by ants (Berg 1969). Soon after the forest canopy is closed, the photosynthetic leaf and the old tuber degrade as the new tuber enters dormancy (C.‐H. Lin, unpublished data; see Appendix S1e), converting sugar into starch over the summer for use in the following spring (Risser & Cottam 1968).

We studied populations at three sites, 26–90 km apart from each other, in central Ohio, United States: Bohannan (N40°21′05″, W082°55′35″), Sharon Woods (N40°07′12″, W082°58′01″; “Sharon” hereafter), and Mathias Grove (N39°35′10″, W082°32′47″; “Mathias” hereafter). The Bohannan and Sharon populations are located in forest remnants surrounded by agricultural and suburban land uses, respectively. The Mathias population is located in a well‐forested landscape. These populations also differed with respect to density and spatial structure (see Appendix S2 for plant density data). The Bohannan population had the lowest population density of all sites, with scattered individuals and very few flowering plants. The Sharon population had a distinctly patchy distribution, with individuals aggregating in discontinuous patches and some isolated individuals between patches. The Mathias population formed large continuous patches in higher density compared to the other sites. We selected sites that encompassed the types of landscapes where *D. canadensis* occurs – from isolated forest remnants to pristine forest, in order to produce generalizable results for the species.

### Field census

#### Vital rates

To record vital rates (e.g., survival, growth, and reproduction) for population modeling, we setup eight 1‐m^2^ quadrats, separated by at least 5 m, at each site in early April of 2008 and monitored *D. canadensis* individuals in quadrats at one‐year intervals for three consecutive years. Plots were selected to capture any spatial variation that may exist at each study site. Frequent browsing by deer in Sharon and Bohannan necessitated the use of deer fences around census plots. Because the quadrats contained very few flowering plants, as proportional to the natural population, we tagged additional 15–20 flowering plants near the census quadrats to monitor more accurately the fates of flowering plants. To maintain the sample size, additional plants were tagged in 2009 and 2010 to replace plants that died or could not be relocated.

Plants in the census plots were monitored for survival, growth, and clonal and sexual reproductive status each year. At the end of each spring, after the photosynthetic leaf had withered, we measured the diameter of the primary tuber produced during the current year and, when present, the diameters of old tubers. The number of seeds per plant and the number and size of secondary tubers produced by tagged plants were recorded to calculate reproductive probabilities. A total of 1701–2030 individuals per site were monitored throughout the census period, resulting in an overall total of 5745 individuals.

#### Seed and tuber germination tests

Separate experiments were conducted to measure seed germination and ramet production from tubers. The probability of seed germination was estimated from seeds that were collected outside the census quadrats and sowed in germination baskets made of metal wire frames and fiberglass window screens. Eight baskets, containing 50 seeds each, were buried under the litter layer near the census quadrats in 2010 at Mathias and Sharon, and were examined for seedlings in 2011. Only four baskets were used at Bohannan because seed production was low in this population. We did not observe any seed dormancy that lasted longer than 1 year in seeds sowed in the year prior to the germination test.

Tuber germination probability was estimated using secondary tubers that had detached from parent plants, collected in the litter layer outside the census quadrats. A total of 342–511 tubers, varying in size, were used from each site. The tubers were sowed loosely in sieved local soil contained in 4–6 germination baskets. Germination baskets were buried under the litter layer near the census quadrats and examined in the following spring to record the number and size of plants developed from secondary tubers. Live tubers (retaining yellow coloration) that did not germinate were considered dormant. We followed the survival, germination, and growth of tubers in the germination baskets for 2 years.

#### Pollination experiments

We conducted pollinator exclusion experiments and supplemental pollination experiments to test whether seed set was limited by inadequate insect pollination, and to investigate how changes in sexual reproductive output may influence clonal reproduction and population growth rates. We randomly chose of 15–25 pairs of “experimental” flowering plants, similarly sized, near the census quadrats. The inflorescence of one experimental plant was covered with fine nylon mesh to exclude pollinators. Flowers on the other experimental plant remained uncovered and were supplemented with pollen from at least two conspecific donors located at 100 m away to maximize pollination success. Flowering plants that did not receive any manipulation were “control” plants with natural levels of pollination. Pollinator exclusion was applied at all sites in 2008 and at Sharon and Mathias in 2009. We could not find a sufficient number of flowering plants for pollination manipulation at Bohannan in 2009, so only natural seed production was monitored. Mean number of seeds per plant was calculated for each treatment at each site and year. Means were compared using ANOVAs. If seeds were limited by inadequate pollination, supplemental pollination would be expected to produce more seeds than the control treatment. Vital rates (size and number of tubers per plant, seeds per plant, and size and number of tubers in the following year if the plant survived) of the experimental plants were also recorded for demographic analyses.

### Data analyses

#### Life cycle diagram

**Figure 1:**
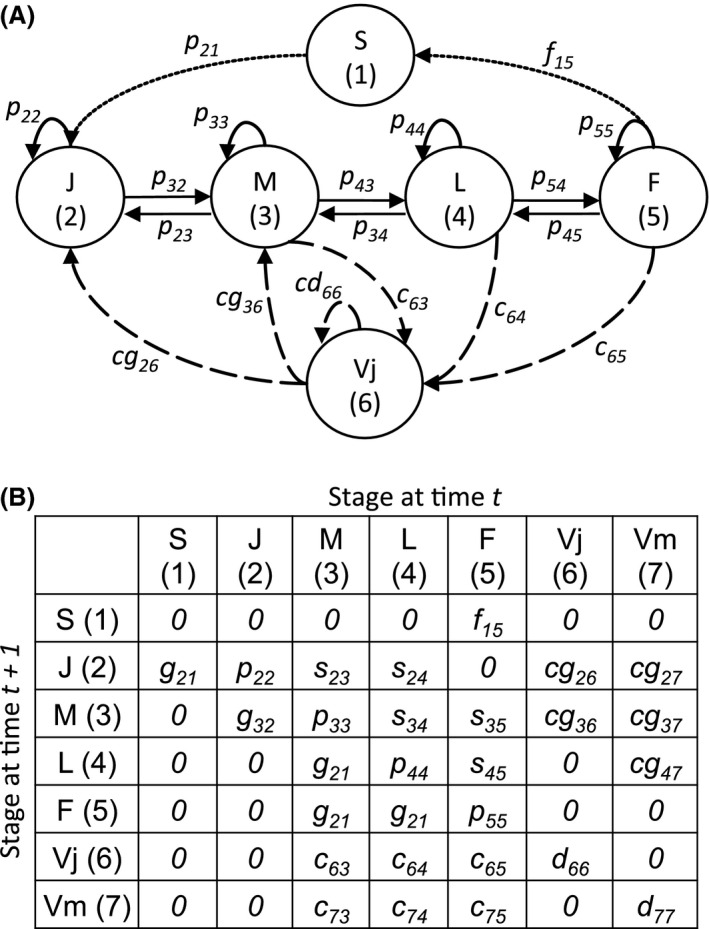
Life cycle graph for *Dicentra canadensis* (A) and the corresponding stage transition matrix (B). Circles in the life cycle graph represent life stages of seedling (S), juvenile (J), medium nonflowering plants (M), large nonflowering plants (L), flowering plants (F), and clonal tubers (Vj and Vm). For clarity purposes, only one of the two tuber stages, Vj, is presented in the life cycle graph. Transition probabilities between stages are presented as arrows, which include the growth, regress, or no change of stage of existing plants (*p*_*ij*_), fecundity of flowering plants (*f*_15_), and the production (*c*_*ij*_), germination (*cg*_*ij*_), and dormancy (*cd*_*ij*_) of clonal tubers. Solid lines represent survival of existing individuals, while dotted and dashed lines represent recruitment via sexual and clonal reproduction, respectively.

Field census data were used to construct a life cycle graph (Fig. 1A) to measure the demographic significance of reproductive pathway at each location. Seven life cycle stages, distinguished by size and reproductive conditions, were identified as follows:

1. S, seedlings, develop from seeds.
2. J, juveniles with primary tubers <4 mm in diameter, similar size as seedlings.
3. M, medium nonflowering plants with primary tubers 4–8 mm in diameter.
4. L, large nonflowering plants with primary tubers >8 mm in diameter.
5. F, flowering plants. Typically, only plants with large tubers can produce flowers although flowering plants with medium‐sized tubers were occasionally observed.
6. Vj, juvenile‐sized secondary tubers, <4 mm in diameter.
7. Vm, medium‐sized secondary tubers, >4 mm in diameter. Large secondary tubers (>8 mm) were occasionally found but rarely dispersed (only 2% of dispersed tubers were >8 mm). Therefore, we grouped all secondary tubers >4 mm in the same stage.

#### Calculation of transition probabilities

Census data were used to estimate the probabilities of transitions between stages corresponding to the life cycle graph (Fig. 1) each year. A plant of a given stage (J, M, L, or F) could remain in the same stage, or transition to a different stage by growth or regression in a year interval; probabilities of such changes, denoted as *p*_*ij*_, were calculated from the census records of tagged plants. Sexual fecundity (*f*_15_), defined as the number of seedlings produced per flowering plant, was calculated as the multiplication product of the number of seeds produced per plant and the germination probability for seeds sowed in germination baskets. Because seedlings produce a primary tuber that is similar to that of a juvenile ramet, we assumed all surviving seedlings moved on to the juvenile stage, and used the survival probability of juveniles for seedlings.

The probability of producing clonal tubers (*c*_*ij*_) was the frequency of secondary tubers that were produced by and detached from a parent plant. An independent clonal tuber, if survived, could either stay dormant as a tuber (*cd*_*ii*_) or grow to a new ramet (*cg*_*ij*_), of which the probabilities were estimated from the tuber germination tests.

#### Demographic analyses

Matrix projection models (Caswell 2001) were constructed to examine the population‐level significance of reproductive pathway for each population and year (Fig. 1B; see Appendix S3 in Supporting Information for transition matrices). The asymptotic growth rate, *λ*, was calculated as the dominant eigenvalue for each transition matrix. The value of *λ* indicates the rate of change of population size on the long term; therefore, *λ *> 1 when a population is growing, *λ *< 1 when a population is declining, and *λ *= 1 when a population maintains a stable size. Because an asymptotic growth rate assumes constant environmental conditions, it is not a realistic indicator for long‐term population growth in a variable environment such as the temperate forest understory (Bierzychudek 1982a; Caswell 2001; Lande et al. 2003). Therefore, *λ* is used here as a proxy for population fitness (Caswell 2001; Sibly and Hone 2002), so that the performance of different populations and under different pollination scenarios can be compared. We used bootstrapping methods with 1000 iterations to calculate a mean *λ* value and 95% confidence intervals around the mean for each matrix.

To address whether the growth of a population relied equally on sexual and clonal reproduction, we performed loop analyses, an extension of the elasticity analysis (van Groenendael et al. 1994), to quantify the relative contributions of clonal and sexual reproductive pathways to *λ*. Elasticity analysis measures the contribution of a demographic process, such as growth or recruitment of a given size class, to *λ* (Caswell 2001). Changes in processes with high elasticities will generate proportionally greater changes in *λ*. Therefore, integrating life stage transitions into unique loops permits quantitative comparison of the relative contributions of different reproductive pathways to *λ* (van Groenendael et al. 1994; Wardle 1998; de Kroon et al. 2000; Guneralp 2007). A population loop describes the path beginning and ending from a given life history stage. A simple example would be a seedling growing through all four ramet size classes and producing at least one seed that germinates and becomes a seedling.

The life cycle of *D. canadensis* was decomposed into unbranched loops such that each transition was incorporated into at least one loop, and each loop contained at least one unique transition. The elasticity of this unique element defines the characteristic elasticity of the loop. Loop elasticity is calculated as its characteristic elasticity multiplied by the number of transition elements in the loop. We used the MATLAB functions published by Guneralp (2007) to generate loops (Appendix S4) and to calculate the total loop elasticity for each. For each matrix, loops were grouped into three categories. *Sexual* loops were characterized by including the transition from a flowering plant to seedling, while *clonal* loops were characterized by including any transition between tubers and ramets. Loops that did not contain transitions involving seedlings or tubers were the *survival* loops, which depicted the survival, growth, or regression of existing ramets. The sums of elasticities of sexual loops and of clonal loops were used to assess the relative contributions of sexual and clonal forms of reproduction to *λ*, respectively, for each population in each year. All matrix computation was performed in MATLAB 7.11.0 (The MathWorks, Inc. Natick, MA).

To simulate the population‐level effects of pollen availability, we substituted vital rates of flowering plants from pollinator exclusion and supplemental pollination experiments with values of the unmanipulated plants, and determined whether lack of pollination or reinforced pollination, respectively, affects *λ*. We then performed 1000 iterations of bootstrap resampling to obtain mean values of *λ*, loop elasticities, and 95% confidence intervals around the means for each population and compared treatment effects.

#### Resource allocation between sexual and clonal reproduction

We evaluated the costs of two components of sexual reproduction with respect to an individual's ability to reproduce clonally: flower production and seed production. The costs were calculated as the difference between flowering plants and large nonflowering plants in (1) the number of secondary tubers produced in the current year and (2) the proportional increment in biomass of the primary tuber between the current and previous year. Final biomass of the primary tuber produced in the current year represents an individual's energy reserve for the following year.

To estimate tuber biomass, we measured the diameters of 250 tubers collected haphazardly from the study populations after the census in 2008. The tubers were oven‐dried in 55°C for 72 h, and the biomass (dry weight) of each individual tuber was recorded. Cubed diameter of fresh tubers was plotted against dry weight to generate a linear regression equation, which was used to calculate dry mass (*W*, mg) from the diameter (*D*, mm) of a fresh tuber:

(1) $$W=0.246\times D^{3}$$

We estimated the percent change in biomass from the previous year's primary tuber (energy reserve for time *t*, denoted by *W*_*t*_) to the new primary tuber (energy reserve for time *t* + 1, denoted by *W*_*t+1*_) as:

(2) $$(\left(W_{t+1}-W_{t}\right)/W_{t})\%$$

ANOVA was performed to compare proportional biomass change of the primary tuber between flowering and large nonflowering plants, with stage (F vs. L) and year as effects for each site. Because the numbers of secondary tubers and seeds produced per individual followed a right‐skewed distribution, nonparametric tests were used to analyze the effect of seeds on the production of secondary tubers and percent biomass increment in primary tubers. Statistical analyses were conducted in JMP 9.0 (SAS Institute Inc., Cary, NC, USA).

## Results

### Variation in sexual and clonal reproduction

**Figure 2:**
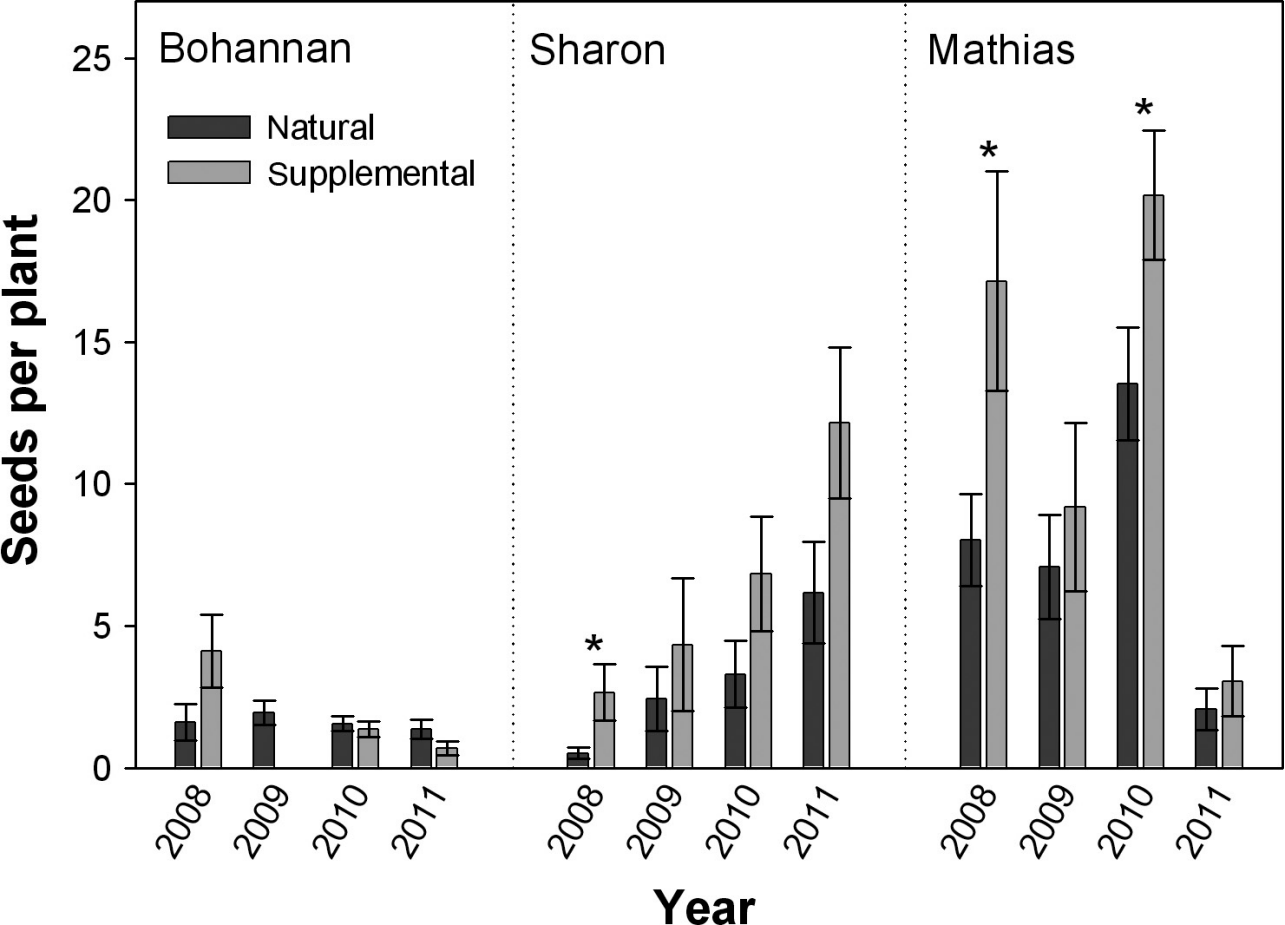
Mean seed set per plant (±SE) in natural and supplemental pollination treatments across three sites in 4 years. Asterisk (*) indicates significant difference (*P* < 0.05) between means of natural versus supplemental seed set in a given site and year. See Appendix S5 for statistical comparison between natural and supplemental pollination at each site per year. Supplemental pollination was not performed at Bohannan in 2009 due to low availability of flowering plants.

Plants produced no seed when pollinators were excluded, confirming that pollinator visits are required for *D. canadensis* to produce seed. In natural and supplemental pollination treatments, seed production varied greatly across sites and years (Fig. 2). The Mathias population generally produced at least three times as many seeds per plant as the Bohannan and Sharon populations for both pollination treatments in 3 of the 4 years (Fig. 2). When seed production at the population level was compared within the natural treatment only, there were significant effects across three sites (ANOVA, *F*_2, 336_ = 21.76, *P *<* *0.0001) and 4 years (*F*_3, 336_ = 3.40, *P *=* *0.018), as well as a significant site × year interaction (*F*_6, 336_ = 6.86, *P *<* *0.0001). This result indicates strong spatial and temporal variation in sexual reproductive output among populations. Similar trends were found in seed set with the supplemental pollination treatment, with which population means varied by site (*F*_2, 181_ = 26.74, *P *<* *0.0001), year (*F*_2, 181_ = 4.02, *P *=* *0.02), and site × year interaction (*F*_4, 181_ = 13.92, *P *<* *0.0001). Significant variation in seed set among years, even when flowers were supplemented with outcross pollen, suggested strong influence of nonpollinator factors on sexual reproduction that varies among years.

Supplemental pollination generally resulted in more seeds than natural pollination, although only at Mathias and Sharon did we find pollen limitation that was statistically significant (Table 1). The Bohannan population had the lowest seed set of all but failed to increase seed production with supplemental pollination. Effect of year was significant for all sites but effect of year × treatment interaction was only observed at Bohannan (Table 1). When each year was analyzed independently, significant pollen limitation was detected only at two sites in 2008 and one site in 2010 (Fig. 2, Appendix S5).

**Table 1:** Summary of ANOVAs showing effects of pollination treatments (natural vs. supplemental), year, and year × treatment interactions on mean seeds per plant. A separate analysis was conducted for each site. Bold values indicate statistical significance (*P* < 0.05)

Site	ANOVA statistics summary		
	df	*F*	*P*
Mathias			
Treatment	1, 194	9.16	**<0.0001**
Year	3, 194	19.24	**0.003**
Year × treatment	3, 194		0.23
Sharon			
Treatment	1, 180	8.29	**0.0045**
Year	3, 180	7.52	**<0.0001**
Year × treatment	3, 180	0.60	0.61
Bohannan^a^			
Treatment	1, 142	1.64	0.20
Year	2, 142	6.06	**0.003**
Year × treatment	2, 142	4.84	**0.009**

a Supplemental pollination was not performed at Bohannan in 2009.

The proportion of large plants (44.5–44.8%, *χ* ^2 ^= 0.01, *P* = 0.9) and flowering plants (95.2–98.0%, *χ* ^2^ = 2.34, *P* = 0.3) that reproduced clonally were similar across populations. However, medium plants reproduced clonally more frequently in Mathias (14.6%) than in the other two populations (9.9% in Bohannan and 10.4% in Sharon; *χ* ^2^ test *P* < 0.0001). Of plants that reproduced clonally, the number of secondary tubers produced per plant varied significantly among years (site‐specific nonparametric Kruskal–Wallis tests, *P* < 0.03 for all comparisons, data not shown) and among sites for flowering and large nonflowering plants (Table 2).

**Table 2:** Summary of secondary tubers produced by clonally reproducing plants. The number of tubers produced by each life stage was compared across sites using nonparametric Kruskal–Wallis tests, with different letters (A, B, or C) denoting significant difference between sites in post hoc pairwise comparisons

Stage	Site	*N*_c_	Mean ± SE	Pairwise	Kruskal–Wallis test
F	Bohannan	159	1.59 ± 0.097	A	*P* < 0.0001
	Sharon	194	2.44 ± 0.123	B	
	Mathias	198	1.85 ± 0.085	C	
L	Bohannan	360	1.38 ± 0.042	A	*P* = 0.013
	Sharon	468	1.70 ± 0.061	B	
	Mathias	313	1.66 ± 0.071	A, B	
M	Bohannan	90	1.09 ± 0.030	n.s.	*P* = 0.065
	Sharon	47	1.21 ± 0.091	n.s.	
	Mathias	92	1.30 ± 0.078	n.s.	

*N*_c_, number of plants producing clonal tubers.

Tuber production in flowering plants that produced seeds (1.98 tubers/plant, SE = 0.078, *N* = 308 plants) did not significantly differ from those without seeds (1.88 tubers/plant, SE = 0.091, *N* = 225; nonparametric Wilcoxon test, *χ* ^2^ = 1.44, *P* = 0.23); therefore, there is no significant effect of seed production on the number of clonal offspring. Pollen supplementation had no effect on the proportion of plants that reproduced clonally (*P* > 0.42 for all site‐specific chi‐square tests comparing treatments).

### Population growth rates

**Figure 3:**
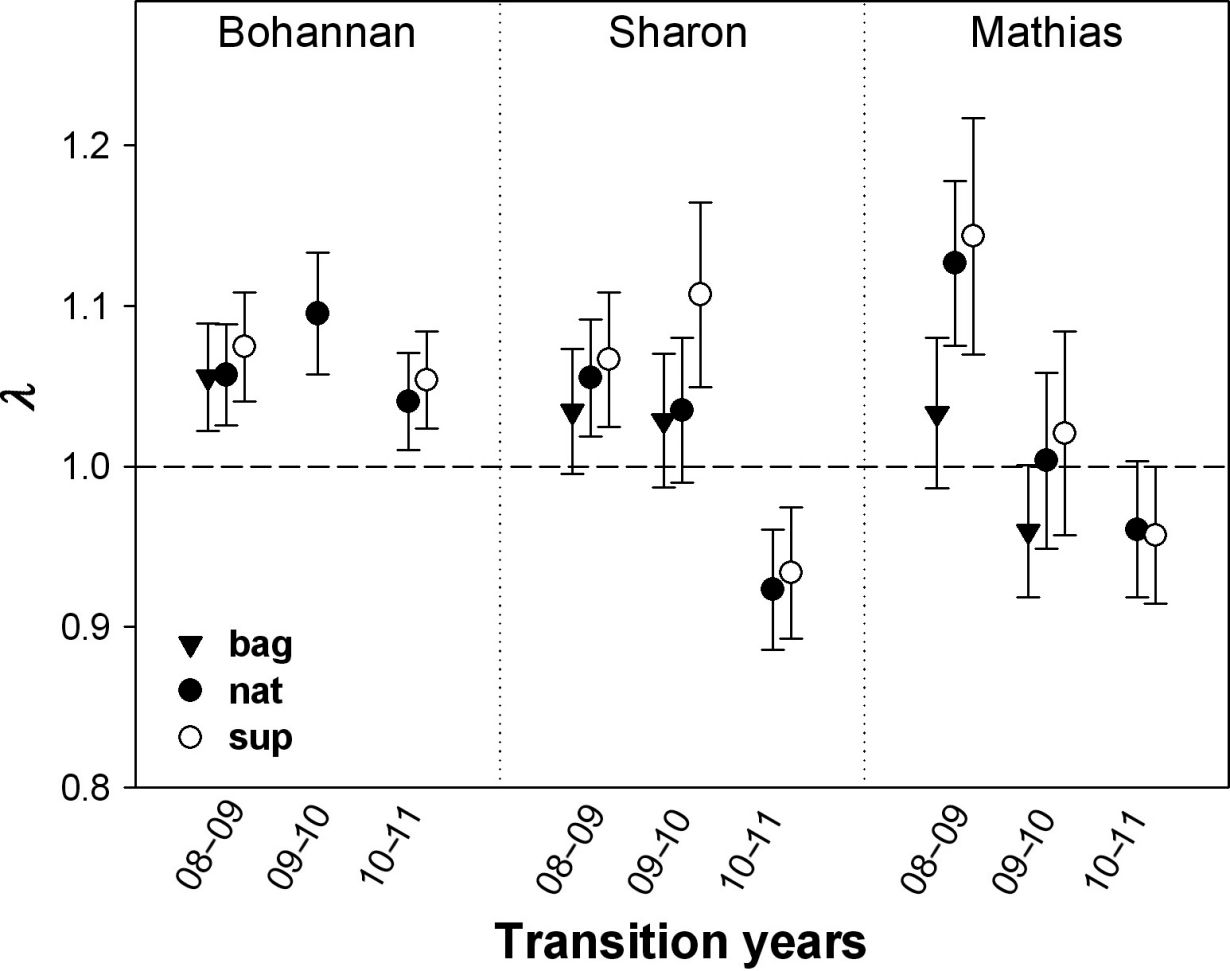
Population growth rates (*λ*) with pollinator exclusion (bag), natural pollination (nat), and supplemental pollination (sup) scenarios. Whiskers are 95% confidence intervals generated with bootstrapping. Pollinator exclusion was only performed in 2008 and 2009 except for Bohannan where only natural pollination was performed in 2009 because flowering plants were scarce that year.

With natural pollination, population growth rates generally ranged between *λ *= 0.9 and *λ *= 1.1, suggesting nearly constant population sizes for all sites and years (Fig. 3). Mean *λ* values at Bohannan were consistently and significantly above *λ *= 1 throughout the census years, but showed significant temporal variation at Sharon and Mathias (Fig. 3). Neither pollinator exclusion nor supplemental pollination treatments significantly affected *λ*, although supplemental pollination slightly increased the means compared to natural pollination in most cases (Fig. 3).

### Loop analyses

**Figure 4:**
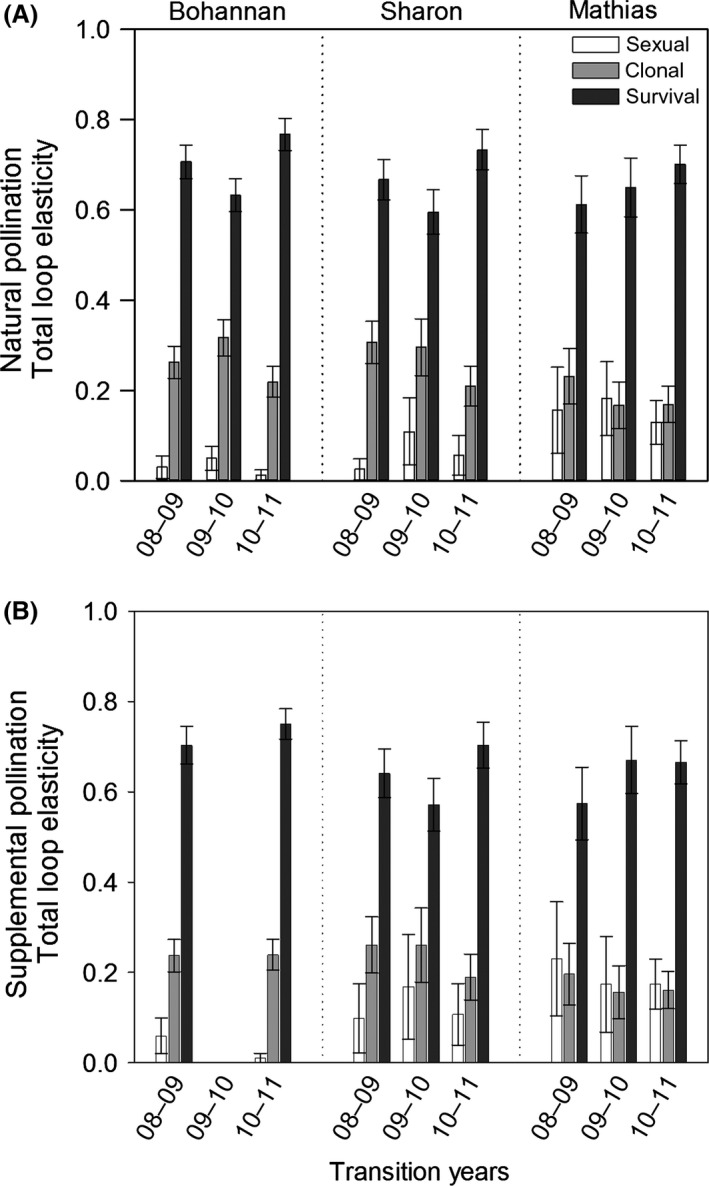
Total loop elasticities of sexual, clonal, and survival loops in populations with (A) natural pollination and (B) supplemental pollination treatments. Whiskers represent 95% confidence intervals around the mean, calculated from 1000 iterations. Supplemental pollination was not performed at Bohannan in 2009.

Loop analyses revealed that the survival loops accounted for more than 60% of population growth rate for all populations (Fig. 4). With natural pollination, elasticities of the clonal loops were significantly higher than sexual loops except at Mathias where we found the highest natural seed set of all populations (Fig. 4A). Modeling population growth with flowering plants receiving supplemental pollination treatments increased the elasticities of population growth rate to sexual reproduction, compared to natural pollination, but did not change the pattern of elasticity distribution among the three pathways (Fig. 4B). At Sharon, supplemental pollination reduced the difference between clonal and sexual pathways such that the confidence intervals overlapped in two of the three transition years (Fig. 4B). Elasticity values of sexual loops never significantly exceeded those of clonal loops even when seed set was enhanced with pollen supplementation.

### Resource allocation

Seed production at the individual level had negligible effects on clonal reproduction and primary energy reserves. Pollination treatments did not significantly affect the size increment of primary tubers or the number of secondary tubers produced in any site or year (Wilcoxon tests, *P* > 0.1 for all sites, data not shown). When data from natural and supplemental pollination were pooled to examine the effect of seed number, no significant correlation was detected between seed number and the size increment of primary tubers or the number of secondary tuber. The only positive correlation was found between seed and tuber production at Mathias in 2008 (Spearmen's *ρ *= 0.40, *P *=* *0.01), reflecting the isometric relationship between number of tuber produced and number of seeds matured.

**Figure 5:**
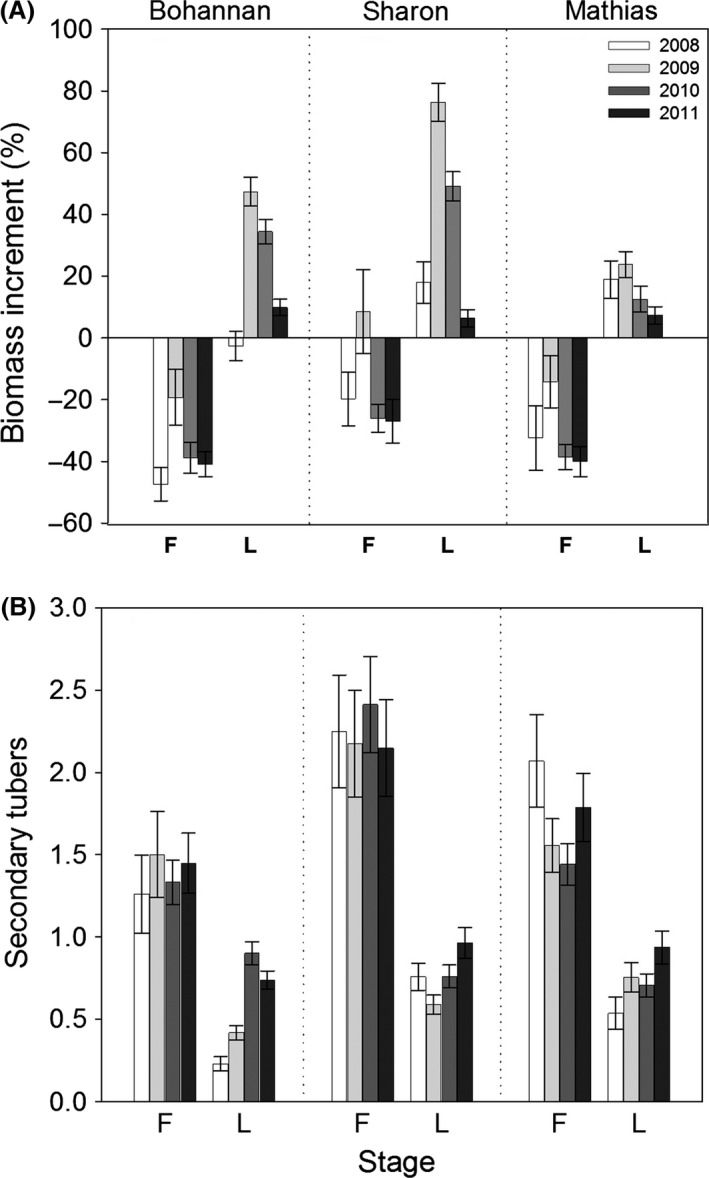
Comparison of flowering (F) versus large nonflowering (L) plants in terms of (A) individual growth, measured as changes in dry biomass of the primary tuber and (B) number of secondary tubers produced per plant. Whiskers represent standard errors around the mean.

The trade‐off between flowering and growth of future energy reserve was evaluated by comparing tuber growth between flowering and large nonflowering plants. Flowering plants generally reduced their energy reserves as indicated by reduced biomass of the primary tuber, whereas large nonflowering plants on average increased the size of primary tubers between years (Fig. 5A, ANOVAs, stage effect *P* < 0.001 for all sites, analysis summary in Appendix S6). Despite their smaller primary tuber size, flowering plants produced on average 0.4–1.6 more secondary tubers than large nonflowering plants (Fig. 5B, independent Wilcoxon tests comparing stages for each site and year, *P* < 0.001 for all tests).

## Discussion

The highly variable sexual reproduction we reported for *D. canadesnsis* typifies the life histories of spring ephemeral plants in the understory of temperate forests (Bierzychudek 1982b; Whigham 2004). Seed production of these plant species may be associated with highly variable pollinator activities in early spring (Schemske et al. 1978; Kudo et al. 2004). Abiotic factors such as temperature, light, or precipitation that are stochastic during the brief reproductive season also may influence sexual reproductive output (McKenna and Houle 2000; Kudo et al. 2008). However, limited sexual reproduction did not have a significant impact on population growth rates of *D. canadensis*. In fact, the population with the lowest average seed set consistently had positive and stable *λ* throughout the census years, indicating the importance of recruitment by clonal reproduction. Populations that produced seeds more frequently showed higher variability in *λ* values but still maintained their growth rates near *λ *= 1. Our analysis using matrix projection models revealed that these populations were maintained by different degrees of dependence on clonal versus sexual reproduction.

Results of loop analysis also showed differential dependence on sexual reproduction among populations, which was associated with variation in seed set. The clonal loops contributed significantly more to *λ* than the sexual loops at the Bohannan population where seed production was the lowest. At the Mathias population, where seed production was high, sexual and clonal loops contributed approximately equally to *λ*. At the Sharon population, where seed production was intermediate, reproduction appeared opportunistic. Here, clonal reproduction was the predominant means of recruitment with natural pollination. When seed set was enhanced with supplemental pollination, the elasticity of sexual reproduction increased so that it was not significantly different from that of clonal reproduction (Fig. 4B). This result suggests that both reproductive modes contributed equally to *λ* when pollen limitation was alleviated. However, sexual reproduction never contributed significantly more than clonal reproduction to *λ* even when the highest seed production was achieved.

Similar conclusions about the overwhelming dependence of populations on survival and clonal reproduction had been reported in other understory forbs (e.g., Nault and Gagnon 1993; Damman and Cain 1998). In *Allium tricoccum*, removal of sexual reproduction from a simulated population model only slightly decelerated population growth, whereas removal of clonal reproduction could lead to population decline (Nault and Gagnon 1993). A 7‐year demographic study also found strong spatial and temporal variation in clonal and sexual reproduction in populations of *Asarum canadense* (Damman and Cain 1998). Although *A. canadense* produces seeds by self‐pollination, which relieves pollen limitation, population growth was still most strongly influenced by clonal growth and survival but insensitive to variation in sexual reproduction (Damman and Cain 1998). The prevalence of clonal reproduction in temperate forest forbs suggests the adaptive significance of this life history trait in understory habitats (Abrahamson 1979; Bierzychudek 1982b).

To some degree, the extent to which the population relies on sexual versus clonal reproduction depends on the fitness of individuals engaging in each strategy. At the individual level, allocating resources to one reproductive strategy reduces the resources available for the other (Thompson and Eckert 2004). We found that plants producing flowers allocated less biomass to primary tubers, thereby reducing their reproduction and survival in the following year. The cost of making seeds was relatively small compared to that for flowers. The photosynthetic pericarps and inflorescence stalks of *D. canadensis* could supply energy to developing seeds (Case and Ashman 2005; Horibata et al. 2007). Maintaining the robust, nectar‐rich flowers of *D. canadensis* for several days appears more energetically expensive than producing seeds.

Contrary to the prediction that allocation of resources to sexual reproduction would diminish the clonal productivity, flowering plants of *D. canadensis* on average produced more tubers than nonflowering plants of similar size (Fig. 5B). This additional allocation of biomass to secondary tubers could be explained by structural constraints of the flowering plant. The flowering plant produces an inflorescence stalk (Appendix S1) that supports not only the flowers, but also the weight of a queen bumble bee hanging upside‐down on the flower while probing for nectar. The stalk is typically situated between the primary tuber and a secondary tuber (almost always the largest one if multiple secondary tubers are present). This tuber structure appears to provide physical support to the flowering stalk. Because the supportive secondary tuber typically degrades with the parent's old tuber instead of becoming a new ramet in the following year, it is essentially an investment for sexual reproduction. Considering the energy demand for flower maintenance along with reduced survival and growth of the individual, flowering is therefore a very costly decision in *D. canadensis*.

Investments by individuals in sexual reproduction could negatively affect survival (Ehrlén and Eriksson 1995) or the quantity and quality of clonal offspring (van Drunen and Dorken 2012). Consequently, *λ* can be negatively impacted if the cost is not offset by successful recruitment of seedlings (Horvitz et al. 2010). As *D. canadensis* has very low seed germination and seedling survival, seed production unlikely contributed enough to offset the cost of flowering to *λ*, even when seed set was enhanced with pollen supplementation. Hence, clonal reproduction may provide a more reliable fitness gain than sexual reproduction and play a more significant role in population growth.

At the population level, clonality may impose density‐dependent stress that interferes with sexual reproduction. As clonal ramets of *D. canadensis* often aggregate in patches, growth and survival of seedlings may be inhibited by intense intraspecific competition (Eriksson 1992). Additionally, pollination success could be discounted by spatial aggregation of clone mates, which increases the frequency of inter‐ramet geitonogamy and further reduces seed set (Charpentier 2002). Although approximately one‐fifth of seeds germinated in the artificial baskets, we very rarely observed natural seedlings in *D. canadensis* patches, suggesting possible intra‐specific competition with established ramets or other density‐dependent effects that increased seedling mortality. Because *D. canadensis* seeds degraded if not germinated in the following year (C.‐H. Lin, unpublished data), it was unlikely that seeds could stay dormant and wait for favorable conditions for germination.

Although this study was not designed to test the influence of habitat quality on reproductive mode, we must note that sexual reproduction occurred more frequently in the population located in continuous forest than those in forest fragments. Fragmentation and disturbance of forest habitats could reduce pollination or exacerbate the variability of sexual reproduction by altering pollinator abundance (Potts et al. 2010; Roulston and Goodell 2011) and microhabitat conditions that affect seed set and seedling survival (Meier et al. 1995; Vandepitte et al. 2009). The lack of seed production in Bohannan, however, was not caused by inadequate pollen delivery, as revealed by the population's insensitivity to supplemental pollination. What, then, could cause such extremely depressed sexual reproduction? Small populations may suffer acute mate limitation if genetic variability is eroded by genetic drift to the point that compatible genotypes are scarce (Wagenius et al. 2007; Pickup and Young 2008; Lin 2013). Severe effects of mate limitation have been reported in other self‐incompatible clonal plants including *Convallaria majalis* (Vandepitte et al. 2010) and *Linnaea borealis* (Scobie and Wilcock 2009). In these plants, reduced genetic variation resulted in monoclonal patches that could not produce seed. Consistent with these studies, genetic analysis of our *D. canadensis* populations revealed a monoclonal population structure at Bohannan and a positive relationship between genet diversity and seed set across populations (Lin 2013), supporting the hypothesis that scarcity of compatible mates inhibited sexual reproduction.

**Figure 6:**
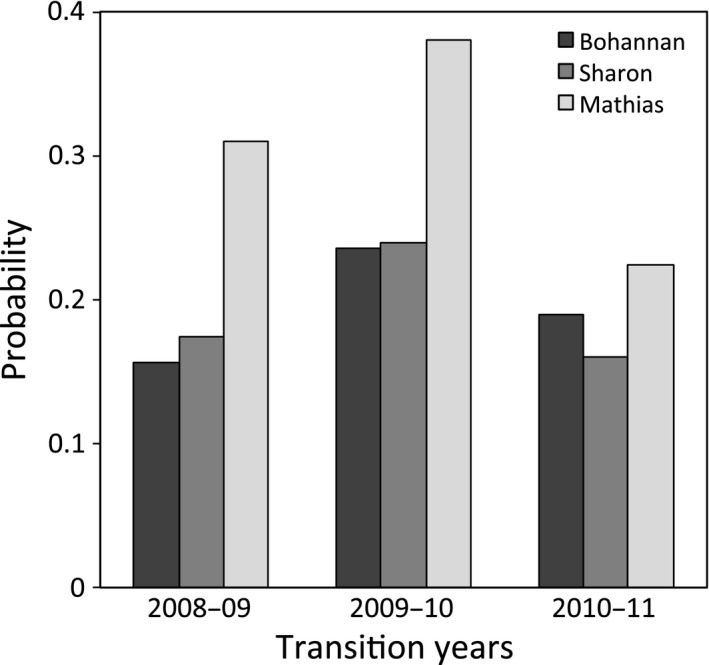
Probabilities of L (large nonflowering) plants transitioning into F (flowering) plants. Infrequent transitions from L to F at Bohannan and Sharon indicated that more adult plants stayed nonflowering in these populations than in the Mathias population.

Our data show that the combined effect of declining habitat quality and genetic degradation disadvantaged flowering individuals. Energy investment in flowers was not returned by successful seedling recruitment, while the individual suffered a loss in growth and clonal reproduction. Prolonged, unsuccessful sexual reproduction can cause the proportion of flowering adults in clonal populations to decline overtime (e.g., *Decodon verticillatus*; Eckert et al. 1999; Dorken and Eckert 2001). We observed lower probability of flowering in *D. canadensis* at the two remnant sites compared to the well‐forested site, where seeds were produced more frequently (Fig. 6). Populations at fragmented sites may have begun to show changes in response to sexual reproduction failure and increased the proportion of clonally reproducing individuals.

This study contributes to the generalization that clonality is a favorable life history trait for maximizing genet fitness in an environment where sexual reproduction is unpredictable (Ceplitis 2001; Vallejo‐Marin et al. 2010). In *D. canadensis,* this is evidenced by (1) the insensitivity of population growth to fluctuations in seed production and (2) low fitness return for the substantial investment in flowering (Vallejo‐Marin et al. 2010). Reduced sexual reproduction resulting from environmental changes (e.g., loss of pollinator services or genetically compatible mates in fragmented habitats) can escalate a population's shift toward a reproductive strategy that is predominately clonal. Because infertile mutations may accumulate in clonal lineages over time (Ally et al. 2010), over‐dependence on clonal reproduction could eventually lead to permanent loss of sex in a population (Eckert 2002; Honnay and Bossuyt 2005), leaving the population vulnerable to environmental changes that require plants to quickly adapt to the new conditions.

## Conflict of Interest

None declared.

## Data Accessibility

1. Transition matrices for population models: uploaded as online supporting information.
2. Tuber and seed data, including pollination treatments: DRYAD entry doi: 10.5061/dryad.j21v2.

## Supporting information


**Appendix S1.** Photographs of *Dicentra canadensis*.
**Appendix S2.** Plant density information for the study populations.
**Appendix S3.** Annual transition matrices of the study populations.
**Appendix S4** List of life cycle loops generated from transition matrices of D. *canadensis*.
**Appendix S5.** Summary of results in pollinator exclusion, natural pollination, and supplemental pollination treatments at three sites across 4 years.
**Appendix S6.** Summary of ANOVA comparing the percent increment in primary tuber mass between flowering and large, nonflowering plants.
