# Supplementary material for: Demographic consequences of greater clonal than sexual reproduction in Dicentra canadensis
Source: Ecol Evol. 2016 May 10;6(12):3871–83. doi: 10.1002/ece3.2163 (PMC4867665; doi:10.1002/ece3.2163)
Supplement: Supplementary file 1 — Appendix S1. Photographs of Dicentra canadensis. Appendix S2. Plant density information for the study populations. Appendix S3. Annual transition matrices of the study populations. Appendix S4 List of life cycle loops generated from transition matrices of D. canadensis. Appendix S5. Summary of results in pollinator exclusion, natural pollination, and supplemental pollination treatments at three sites across 4 years. Appendix S6. Summary of ANOVA comparing the percent increment in primary tuber mass between flowering and large, nonflowering plants. [file ECE3-6-3871-s001.docx]

**Appendix S1.** Photographs of *Dicentra canadensis*. (a) A primary tuber produces a photosynthetic leaf in early spring. (b) View of tubers from the previous year (dark yellow, arrow “p”) and new tubers (pale yellow, “c”) connected with a rhizome in a flowering plant (b) and a large non-flowering plant (c). A flowering plant produces one inflorescence (arrow “i”) that holds several flowers (d). After canopy closure, all aboveground stems wither and tubers produced in the current year turn into a bright yellow color as the plant enters dormancy (e).

**
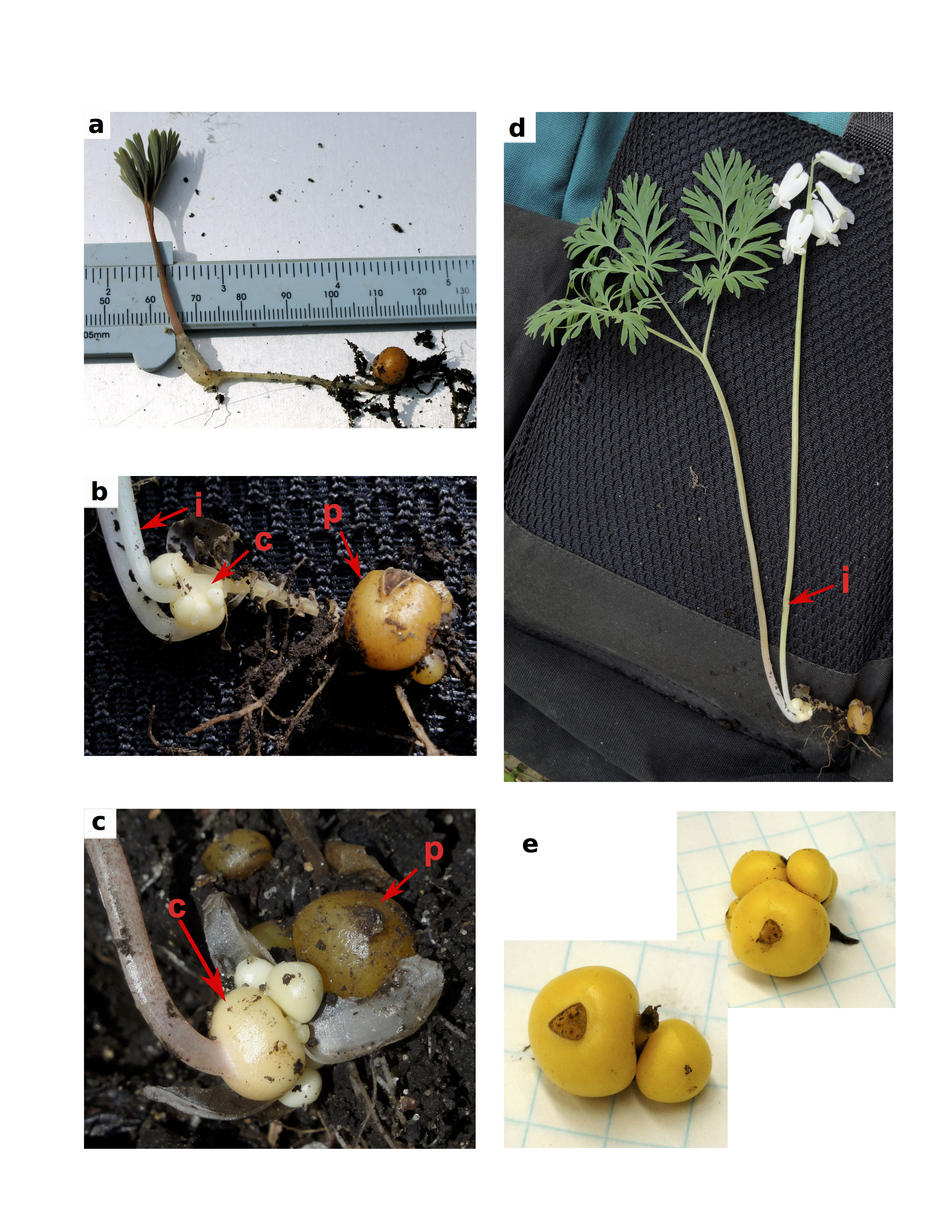
**

**Appendix S2.**  *Dicentra canadensis* plant density of the three study populations. Plant density was measured along two 50 m transects crossed perpendicularly at mid-point. Quadrants (1-m^2^) were placed at 5m intervals along each of the four axes of the cross. All plants of each stage in the quadrats were counted. Data was recorded during the flowering season in 2008.

|  |  | Plants/m2 (s.d.) | | | | |
| --- | --- | --- | --- | --- | --- | --- |
| Site | n* | Juvenile | Medium | Large | Flowering | All stages |
| Bohannan | 21 | 8.5 (13.1) | 30.4 (46.3) | 3.9 (5.1) | 0.4 (0.7) | 43.2 (55.3) |
| Sharon | 21 | 5.4 (5.2) | 46.9 (44.8) | 22.8 (30.0) | 2.0 (2.9) | 77.0 (71.8) |
| Mathias | 18 | 10.9 (6.7) | 75.4 (32.9) | 39.3 (23.3) | 4.7 (5.0) | 130.3 (51.5) |

*n : number of quadrats

**Appendix S3.** Annual transition matrices with flowering plants receiving pollinator exclusion (Bag), natural pollination (Nat), and supplemental pollination (Sup) respectively for three study populations of *D. canadensis.*

| **Bohanna, Bag 2008-2009** | | |  |  |  |  |  |
| --- | --- | --- | --- | --- | --- | --- | --- |
|  | S | J | M | L | F | Vj | Vm |
| S | 0 | 0 | 0 | 0 | 0 | 0 | 0 |
| J | 0.8077 | 0.5385 | 0.0138 | 0 | 0 | 0.1818 | 0.0407 |
| M | 0 | 0.2692 | 0.6912 | 0.0781 | 0.0769 | 0.2273 | 0.2743 |
| L | 0 | 0 | 0.2074 | 0.7188 | 0.7692 | 0 | 0.0031 |
| F | 0 | 0 | 0.0046 | 0.1562 | 0 | 0 | 0 |
| Vj | 0 | 0 | 0.0092 | 0.0078 | 0 | 0.1818 | 0 |
| Vm | 0 | 0 | 0.0968 | 0.6875 | 0.3077 | 0 | 0.1719 |
|  |  |  |  |  |  |  |  |
| **Bohannan, Nat 2008-2009** | | |  |  |  |  |  |
|  | S | J | M | L | F | Vj | Vm |
| S | 0 | 0 | 0 | 0 | 0.37 | 0 | 0 |
| J | 0.8077 | 0.5385 | 0.0138 | 0 | 0 | 0.1818 | 0.0407 |
| M | 0 | 0.2692 | 0.6912 | 0.0781 | 0.2609 | 0.2273 | 0.2743 |
| L | 0 | 0 | 0.2074 | 0.7188 | 0.5652 | 0 | 0.0031 |
| F | 0 | 0 | 0.0046 | 0.1562 | 0 | 0 | 0 |
| Vj | 0 | 0 | 0.0092 | 0.0078 | 0 | 0.1818 | 0 |
| Vm | 0 | 0 | 0.0968 | 0.6875 | 0.4348 | 0 | 0.1719 |
|  |  |  |  |  |  |  |  |
| **Bohannan, Nat 2009-2010** | | |  |  |  |  |  |
|  | S | J | M | L | F | Vj | Vm |
| S | 0 | 0 | 0 | 0 | 0.4819 | 0 | 0 |
| J | 0.8519 | 0.4444 | 0.0262 | 0.0103 | 0 | 0.1818 | 0.0407 |
| M | 0 | 0.4074 | 0.6754 | 0.0718 | 0.1905 | 0.2273 | 0.2743 |
| L | 0 | 0 | 0.1518 | 0.6205 | 0.5714 | 0 | 0.0031 |
| F | 0 | 0 | 0 | 0.2359 | 0.2381 | 0 | 0 |
| Vj | 0 | 0 | 0.0419 | 0.1179 | 0.1429 | 0.1818 | 0 |
| Vm | 0 | 0 | 0.178 | 1.0462 | 0.9524 | 0 | 0.1719 |
|  |  |  |  |  |  |  |  |
| **Bohannan, Nat 2010-2011** | | |  |  |  |  |  |
|  | S | J | M | L | F | Vj | Vm |
| S | 0 | 0 | 0 | 0 | 0.371 | 0 | 0 |
| J | 0.8125 | 0.7188 | 0.0264 | 0 | 0 | 0.1818 | 0.0407 |
| M | 0 | 0.0938 | 0.815 | 0.1555 | 0.2258 | 0.2273 | 0.2743 |
| L | 0 | 0 | 0.0969 | 0.6134 | 0.6774 | 0 | 0.0031 |
| F | 0 | 0 | 0 | 0.1891 | 0.0645 | 0 | 0 |
| Vj | 0 | 0 | 0.0264 | 0.0462 | 0 | 0.1818 | 0 |
| Vm | 0 | 0 | 0.0925 | 0.8235 | 0.5806 | 0 | 0.1719 |
|  |  |  |  |  |  |  |  |
| **Bohannan, Sup 2008-2009** | | |  |  |  |  |  |
|  | S | J | M | L | F | Vj | Vm |
| S | 0 | 0 | 0 | 0 | 0.8127 | 0 | 0 |
| J | 0.8077 | 0.5385 | 0.0138 | 0 | 0 | 0.1818 | 0.0407 |
| M | 0 | 0.2692 | 0.6912 | 0.0781 | 0.1333 | 0.2273 | 0.2743 |
| L | 0 | 0 | 0.2074 | 0.7188 | 0.8 | 0 | 0.0031 |
| F | 0 | 0 | 0.0046 | 0.1562 | 0 | 0 | 0 |
| Vj | 0 | 0 | 0.0092 | 0.0078 | 0 | 0.1818 | 0 |
| Vm | 0 | 0 | 0.0968 | 0.6875 | 0.2667 | 0 | 0.1719 |
|  |  |  |  |  |  |  |  |
| **Bohannan, Sup 2010-2011** | | |  |  |  |  |  |
|  | S | J | M | L | F | Vj | Vm |
| S | 0 | 0 | 0 | 0 | 0.3345 | 0 | 0 |
| J | 0.8125 | 0.7188 | 0.0264 | 0 | 0 | 0.1818 | 0.0407 |
| M | 0 | 0.0938 | 0.815 | 0.1555 | 0.0909 | 0.2273 | 0.2743 |
| L | 0 | 0 | 0.0969 | 0.6134 | 0.8182 | 0 | 0.0031 |
| F | 0 | 0 | 0 | 0.1891 | 0.0909 | 0 | 0 |
| Vj | 0 | 0 | 0.0264 | 0.0462 | 0.0455 | 0.1818 | 0 |
| Vm | 0 | 0 | 0.0925 | 0.8235 | 1.2727 | 0 | 0.1719 |
|  |  |  |  |  |  |  |  |
| **Sharon, Bag 2008-2009** | | |  |  |  |  |  |
|  | S | J | M | L | F | Vj | Vm |
| S | 0 | 0 | 0 | 0 | 0 | 0 | 0 |
| J | 0.8 | 0.4667 | 0 | 0.0097 | 0 | 0.1288 | 0.0099 |
| M | 0 | 0.3333 | 0.5956 | 0.0825 | 0 | 0.1567 | 0.1379 |
| L | 0 | 0 | 0.3388 | 0.6845 | 0.6667 | 0 | 0.0056 |
| F | 0 | 0 | 0 | 0.1748 | 0 | 0 | 0 |
| Vj | 0 | 0 | 0.0055 | 0.0437 | 0.1111 | 0.2543 | 0 |
| Vm | 0 | 0 | 0.0874 | 0.9854 | 0.1111 | 0 | 0.2031 |
|  |  |  |  |  |  |  |  |
| **Sharon, Bag 2009-2010** | | |  |  |  |  |  |
|  | S | J | M | L | F | Vj | Vm |
| S | 0 | 0 | 0 | 0 | 0 | 0 | 0 |
| J | 0.8696 | 0.4783 | 0.0199 | 0.0038 | 0 | 0.1288 | 0.0099 |
| M | 0 | 0.3913 | 0.5563 | 0.0342 | 0.1667 | 0.1567 | 0.1379 |
| L | 0 | 0 | 0.3245 | 0.616 | 0.5833 | 0 | 0.0056 |
| F | 0 | 0 | 0 | 0.2395 | 0.0833 | 0 | 0 |
| Vj | 0 | 0 | 0 | 0.0456 | 0.1667 | 0.2543 | 0 |
| Vm | 0 | 0 | 0.0795 | 1.0837 | 1.1667 | 0 | 0.2031 |
|  |  |  |  |  |  |  |  |
| **Sharon, Nat, 2008-2009** | | |  |  |  |  |  |
|  | S | J | M | L | F | Vj | Vm |
| S | 0 | 0 | 0 | 0 | 0.143 | 0 | 0 |
| J | 0.8 | 0.4667 | 0 | 0.0097 | 0 | 0.1288 | 0.0099 |
| M | 0 | 0.3333 | 0.5956 | 0.0825 | 0.1 | 0.1567 | 0.1379 |
| L | 0 | 0 | 0.3388 | 0.6845 | 0.35 | 0 | 0.0056 |
| F | 0 | 0 | 0 | 0.1748 | 0.3 | 0 | 0 |
| Vj | 0 | 0 | 0.0055 | 0.0437 | 0 | 0.2543 | 0 |
| Vm | 0 | 0 | 0.0874 | 0.9854 | 1.35 | 0 | 0.2031 |
| **Sharon, Nat 2009-2010** | | |  |  |  |  |  |
|  | S | J | M | L | F | Vj | Vm |
| S | 0 | 0 | 0 | 0 | 0.52 | 0 | 0 |
| J | 0.8696 | 0.4783 | 0.0199 | 0.0038 | 0 | 0.1288 | 0.0099 |
| M | 0 | 0.3913 | 0.5563 | 0.0342 | 0.0435 | 0.1567 | 0.1379 |
| L | 0 | 0 | 0.3245 | 0.616 | 0.5652 | 0 | 0.0056 |
| F | 0 | 0 | 0 | 0.2395 | 0.0435 | 0 | 0 |
| Vj | 0 | 0 | 0 | 0.0456 | 0 | 0.2543 | 0 |
| Vm | 0 | 0 | 0.0795 | 1.0837 | 1.2174 | 0 | 0.2031 |
|  |  |  |  |  |  |  |  |
| **Sharon, Nat 2010-2011** | | |  |  |  |  |  |
|  | S | J | M | L | F | Vj | Vm |
| S | 0 | 0 | 0 | 0 | 0.7302 | 0 | 0 |
| J | 0.7333 | 0.5333 | 0.0116 | 0 | 0 | 0.1288 | 0.0099 |
| M | 0 | 0.2 | 0.6012 | 0.0674 | 0.1915 | 0.1567 | 0.1379 |
| L | 0 | 0 | 0.1272 | 0.6241 | 0.7021 | 0 | 0.0056 |
| F | 0 | 0 | 0 | 0.1596 | 0.0213 | 0 | 0 |
| Vj | 0 | 0 | 0.0058 | 0.0248 | 0.0213 | 0.2543 | 0 |
| Vm | 0 | 0 | 0.052 | 0.9539 | 0.9362 | 0 | 0.2031 |
|  |  |  |  |  |  |  |  |
| **Sharon, Sup 2008-2009** | | |  |  |  |  |  |
|  | S | J | M | L | F | Vj | Vm |
| S | 0 | 0 | 0 | 0 | 0.845 | 0 | 0 |
| J | 0.8 | 0.4667 | 0 | 0.0097 | 0 | 0.1288 | 0.0099 |
| M | 0 | 0.3333 | 0.5956 | 0.0825 | 0 | 0.1567 | 0.1379 |
| L | 0 | 0 | 0.3388 | 0.6845 | 0.5833 | 0 | 0.0056 |
| F | 0 | 0 | 0 | 0.1748 | 0.0833 | 0 | 0 |
| Vj | 0 | 0 | 0.0055 | 0.0437 | 0.0833 | 0.2543 | 0 |
| Vm | 0 | 0 | 0.0874 | 0.9854 | 0.9167 | 0 | 0.2031 |
|  |  |  |  |  |  |  |  |
| **Sharon, Sup 2009-2010** | | |  |  |  |  |  |
|  | S | J | M | L | F | Vj | Vm |
| S | 0 | 0 | 0 | 0 | 1.2291 | 0 | 0 |
| J | 0.8696 | 0.4783 | 0.0199 | 0.0038 | 0 | 0.1288 | 0.0099 |
| M | 0 | 0.3913 | 0.5563 | 0.0342 | 0 | 0.1567 | 0.1379 |
| L | 0 | 0 | 0.3245 | 0.616 | 0.7273 | 0 | 0.0056 |
| F | 0 | 0 | 0 | 0.2395 | 0.1818 | 0 | 0 |
| Vj | 0 | 0 | 0 | 0.0456 | 0.0909 | 0.2543 | 0 |
| Vm | 0 | 0 | 0.0795 | 1.0837 | 2.2727 | 0 | 0.2031 |
|  |  |  |  |  |  |  |  |
| **Sharon, Sup 2010-2011** | | |  |  |  |  |  |
|  | S | J | M | L | F | Vj | Vm |
| S | 0 | 0 | 0 | 0 | 1.573 | 0 | 0 |
| J | 0.7333 | 0.5333 | 0.0116 | 0 | 0 | 0.1288 | 0.0099 |
| M | 0 | 0.2 | 0.6012 | 0.0674 | 0.1 | 0.1567 | 0.1379 |
| L | 0 | 0 | 0.1272 | 0.6241 | 0.75 | 0 | 0.0056 |
| F | 0 | 0 | 0 | 0.1596 | 0 | 0 | 0 |
| Vj | 0 | 0 | 0.0058 | 0.0248 | 0.05 | 0.2543 | 0 |
| Vm | 0 | 0 | 0.052 | 0.9539 | 0.9 | 0 | 0.2031 |
| **Mathias, Bag 2008-2009** | | |  |  |  |  |  |
|  | S | J | M | L | F | Vj | Vm |
| S | 0 | 0 | 0 | 0 | 0 | 0 | 0 |
| J | 0.8182 | 0.5455 | 0.0102 | 0 | 0 | 0.0442 | 0.0185 |
| M | 0 | 0.2727 | 0.6939 | 0.0635 | 0.2727 | 0.0442 | 0.1101 |
| L | 0 | 0 | 0.1939 | 0.5794 | 0.2727 | 0 | 0.0264 |
| F | 0 | 0 | 0.0408 | 0.3095 | 0.0909 | 0 | 0 |
| Vj | 0 | 0 | 0.0714 | 0.0952 | 0 | 0.5172 | 0 |
| Vm | 0 | 0 | 0.2143 | 1.2381 | 0.4545 | 0 | 0.3097 |
|  |  |  |  |  |  |  |  |
| **Mathias, Bag 2009-2010** | | |  |  |  |  |  |
|  | S | J | M | L | F | Vj | Vm |
| S | 0 | 0 | 0 | 0 | 0 | 0 | 0 |
| J | 0.875 | 0.625 | 0.0368 | 0.0058 | 0 | 0.0442 | 0.0185 |
| M | 0 | 0.25 | 0.6691 | 0.0877 | 0.3 | 0.0442 | 0.1101 |
| L | 0 | 0 | 0.1324 | 0.4386 | 0.6 | 0 | 0.0264 |
| F | 0 | 0 | 0 | 0.3801 | 0 | 0 | 0 |
| Vj | 0 | 0 | 0.0147 | 0.0877 | 0 | 0.5172 | 0 |
| Vm | 0 | 0 | 0.0809 | 0.8655 | 0.4 | 0 | 0.3097 |
|  |  |  |  |  |  |  |  |
| **Mathias, Nat 2008-2009** | | |  |  |  |  |  |
|  | S | J | M | L | F | Vj | Vm |
| S | 0 | 0 | 0 | 0 | 1.3934 | 0 | 0 |
| J | 0.8182 | 0.5455 | 0.0102 | 0 | 0 | 0.0442 | 0.0185 |
| M | 0 | 0.2727 | 0.6939 | 0.0635 | 0.1667 | 0.0442 | 0.1101 |
| L | 0 | 0 | 0.1939 | 0.5794 | 0.5833 | 0 | 0.0264 |
| F | 0 | 0 | 0.0408 | 0.3095 | 0.1667 | 0 | 0 |
| Vj | 0 | 0 | 0.0714 | 0.0952 | 0.0417 | 0.5172 | 0 |
| Vm | 0 | 0 | 0.2143 | 1.2381 | 0.75 | 0 | 0.3097 |
|  |  |  |  |  |  |  |  |
| **Mathias, Nat 2009-2010** | | |  |  |  |  |  |
|  | S | J | M | L | F | Vj | Vm |
| S | 0 | 0 | 0 | 0 | 1.159 | 0 | 0 |
| J | 0.875 | 0.625 | 0.0368 | 0.0058 | 0 | 0.0442 | 0.0185 |
| M | 0 | 0.25 | 0.6691 | 0.0877 | 0.1154 | 0.0442 | 0.1101 |
| L | 0 | 0 | 0.1324 | 0.4386 | 0.5385 | 0 | 0.0264 |
| F | 0 | 0 | 0 | 0.3801 | 0.1154 | 0 | 0 |
| Vj | 0 | 0 | 0.0147 | 0.0877 | 0 | 0.5172 | 0 |
| Vm | 0 | 0 | 0.0809 | 0.8655 | 0.6538 | 0 | 0.3097 |
|  |  |  |  |  |  |  |  |
| **Mathias, Nat 2010-2011** | | |  |  |  |  |  |
|  | S | J | M | L | F | Vj | Vm |
| S | 0 | 0 | 0 | 0 | 2.4419 | 0 | 0 |
| J | 0.8036 | 0.6607 | 0.0172 | 0.005 | 0 | 0.0442 | 0.0185 |
| M | 0 | 0.1429 | 0.7184 | 0.1095 | 0.2105 | 0.0442 | 0.1101 |
| L | 0 | 0 | 0.0632 | 0.5622 | 0.6316 | 0 | 0.0264 |
| F | 0 | 0 | 0 | 0.2239 | 0.0526 | 0 | 0 |
| Vj | 0 | 0 | 0.0172 | 0.1542 | 0.0789 | 0.5172 | 0 |
| Vm | 0 | 0 | 0.0977 | 1.0149 | 0.4474 | 0 | 0.3097 |
| **Mathias, Sup 2008-2009** | | |  |  |  |  |  |
|  | S | J | M | L | F | Vj | Vm |
| S | 0 | 0 | 0 | 0 | 2.9706 | 0 | 0 |
| J | 0.8182 | 0.5455 | 0.0102 | 0 | 0 | 0.0442 | 0.0185 |
| M | 0 | 0.2727 | 0.6939 | 0.0635 | 0.1667 | 0.0442 | 0.1101 |
| L | 0 | 0 | 0.1939 | 0.5794 | 0.6667 | 0 | 0.0264 |
| F | 0 | 0 | 0.0408 | 0.3095 | 0 | 0 | 0 |
| Vj | 0 | 0 | 0.0714 | 0.0952 | 0 | 0.5172 | 0 |
| Vm | 0 | 0 | 0.2143 | 1.2381 | 0.5833 | 0 | 0.3097 |
|  |  |  |  |  |  |  |  |
| **Mathias, Sup 2009-2010** | | |  |  |  |  |  |
|  | S | J | M | L | F | Vj | Vm |
| S | 0 | 0 | 0 | 0 | 1.3125 | 0 | 0 |
| J | 0.875 | 0.625 | 0.0368 | 0.0058 | 0 | 0.0442 | 0.0185 |
| M | 0 | 0.25 | 0.6691 | 0.0877 | 0 | 0.0442 | 0.1101 |
| L | 0 | 0 | 0.1324 | 0.4386 | 0.8571 | 0 | 0.0264 |
| F | 0 | 0 | 0 | 0.3801 | 0 | 0 | 0 |
| Vj | 0 | 0 | 0.0147 | 0.0877 | 0 | 0.5172 | 0 |
| Vm | 0 | 0 | 0.0809 | 0.8655 | 0.4286 | 0 | 0.3097 |
|  |  |  |  |  |  |  |  |
| **Mathias, Sup 2010-2011** | | |  |  |  |  |  |
|  | S | J | M | L | F | Vj | Vm |
| S | 0 | 0 | 0 | 0 | 3.7951 | 0 | 0 |
| J | 0.8036 | 0.6607 | 0.0172 | 0.005 | 0 | 0.0442 | 0.0185 |
| M | 0 | 0.1429 | 0.7184 | 0.1095 | 0.3077 | 0.0442 | 0.1101 |
| L | 0 | 0 | 0.0632 | 0.5622 | 0.4231 | 0 | 0.0264 |
| F | 0 | 0 | 0 | 0.2239 | 0 | 0 | 0 |
| Vj | 0 | 0 | 0.0172 | 0.1542 | 0.1154 | 0.5172 | 0 |
| Vm | 0 | 0 | 0.0977 | 1.0149 | 0.5769 | 0 | 0.3097 |

**Appendix S4**. List of life-cycle loops generated from transition matrices of *D. canadensis.*

|  |  |
| --- | --- |
| **Loop type** | **Loops** |
| Survival | J --> J |
| Survival | M --> M |
| Survival | L --> L |
| Survival | F --> F |
| Clonal | VJ --> VJ |
| Clonal | VM --> VM |
| Survival | J --> M --> J |
| Survival | M --> L --> M |
| Survival | M --> F --> M |
| Clonal | M --> VJ --> M |
| Clonal | M --> VM --> M |
| Survival | L --> F --> L |
| Clonal | L --> VM --> L |
| Survival | J --> M --> L --> J |
| Survival | M --> L --> F --> M |
| Clonal | M --> L --> VJ --> M |
| Clonal | M --> F --> VJ --> M |
| Clonal | M --> L --> VM --> M |
| Clonal | F --> VM --> M --> F |
| Sexual | S --> J --> M --> F --> S |
| Clonal | J --> M --> L --> VJ --> J |
| Clonal | M --> L --> F --> VM --> M |
| Clonal | F --> VJ --> J --> M --> F |
| Clonal | J --> M --> F --> VM --> J |
| Sexual | S --> J --> M --> L --> F --> S |
| Clonal | J --> M --> L --> F --> VJ --> J |
| Clonal | J --> M --> L --> F --> VM --> J |

**Appendix S5.** Summary of results in pollinator exclusion (bagged), natural pollination, and supplemental pollination treatments at three sites across four years. Seed set resulted from pollination treatments are presented as Mean seeds per plant ± 1 standard error, with sample size (number of plants) in parentheses. The ANOVA results compare means between natural and supplemental pollination treatments in a given site and year.

|  | **Pollination treatments**  **(mean seeds/plant** ± s.e. **)** | | |  | **ANOVA comparing**  **Nat vs. Sup** | |
| --- | --- | --- | --- | --- | --- | --- |
| Year flowered | Bagged | Natural | Supplemental |  | F ratio | P |
| 2008 |  |  |  |  |  |  |
| Bohannan | 0  (13) | 1.61 ± 0.64  (23) | 4.11 ± 1.27  (18) |  | 3.53 | 0.067 |
| Sharon | 0  (18) | 0.52 ± 0.20  (21) | 2.65 ± 1.00  (20) |  | 4.59 | ***0.039*** |
| Mathias | 0  (14) | 8.03 ± 1.61  (29) | 17.1 ± 3.86  (14) |  | 6.66 | ***0.014*** |
| 2009 |  |  |  |  |  |  |
| Bohannan | N/A | 1.96 ± 0.43  (23) | N/A |  | N/A | N/A |
| Sharon | 0  (12) | 2.42 ± 1.13  (19) | 4.33 ± 2.34  (12) |  | 0.67 | 0.419 |
| Mathias | 0  (12) | 6.82 ± 1.80  (28) | 9.18 ± 2.96  (11) |  | 0.48 | 0.493 |
| 2010 |  |  |  |  |  |  |
| Bohannan | N/A | 1.56 ± 0.25  (34) | 1.36 ± 0.28  (25) |  | 0.28 | 0.599 |
| Sharon | N/A | 2.93 ± 1.06  (45) | 6.83 ± 2.02  (23) |  | 3.57 | 0.063 |
| Mathias | N/A | 13.5 ± 1.98  (43) | 20.2 ± 2.28  (29) |  | 4.72 | ***0.033*** |
| 2011 |  |  |  |  |  |  |
| Bohannan | N/A | 1.36 ± 0.33  (28) | 0.7 ± 0.25  (20) |  | 2.17 | 0.147 |
| Sharon | N/A | 6.8 ± 1.78  (28) | 12.2 ± 2.65  (20) |  | 3.77 | 0.058 |
| Mathias | N/A | 2.07 ± 0.73  (27) | 3.05 ± 1.24  (21) |  | 0.5 | 0.482 |

**Appendix S6**. Summary of ANOVA comparing the percent increment in primary tuber mass between flowering and large, non-flowering plants.

| **Site** | df | F | P |
| --- | --- | --- | --- |
| **Bohannan** |  |  |  |
| stage (F *vs.* L) | 1, 704 | 133.02 | <.0001 |
| year | 3, 704 | 9.93 | <.0001 |
| year × stage | 3, 704 | 1.73 | 0.16 |
|  |  |  |  |
| **Sharon** |  |  |  |
| stage(F *vs.* L) | 1, 936 | 56.13 | <.0001 |
| year | 3, 936 | 9.21 | <.0001 |
| Year × stage | 3, 936 | 2.49 | 0.06 |
|  |  |  |  |
| **Mathias** |  |  |  |
| stage (F *vs.* L) | 1, 560 | 62.72 | <.0001 |
| year | 3, 560 | 3.11 | 0.026 |
| Year × stage | 3, 560 | 0.57 | 0.63 |
